# Deep learning diagnostics for bladder tumor identification and grade prediction using RGB method

**DOI:** 10.1038/s41598-022-22797-7

**Published:** 2022-10-21

**Authors:** Jeong Woo Yoo, Kyo Chul Koo, Byung Ha Chung, Sang Yeop Baek, Su Jin Lee, Kyu Hong Park, Kwang Suk Lee

**Affiliations:** 1 Department of Urology, Gangnam Severance Hospital, Yonsei University College of Medicine, 211 Eonju-Ro, Gangnam-Gu, Seoul, 06273 Republic of Korea; 2 Infinyx Corporation, Daegu, Republic of Korea

**Keywords:** Bladder, Urological cancer, Urological cancer

## Abstract

We evaluate the diagnostic performance of deep learning artificial intelligence (AI) for bladder cancer, which used white-light images (WLIs) and narrow-band images, and tumor grade prediction of AI based on tumor color using the red/green/blue (RGB) method. This retrospective study analyzed 10,991 cystoscopic images of suspicious bladder tumors using a mask region-based convolutional neural network with a ResNeXt-101-32 × 8d-FPN backbone. The diagnostic performance of AI was evaluated by calculating sensitivity, specificity, and diagnostic accuracy, and its ability to detect cancers was investigated using the dice score coefficient (DSC). Using the support vector machine model, we analyzed differences in tumor colors according to tumor grade using the RGB method. The sensitivity, specificity, diagnostic accuracy and DSC of AI were 95.0%, 93.7%, 94.1% and 74.7%. In WLIs, there were differences in red and blue values according to tumor grade (*p* < 0.001). According to the average RGB value, the performance was ≥ 98% for the diagnosis of benign vs. low-and high-grade tumors using WLIs and > 90% for the diagnosis of chronic non-specific inflammation vs. carcinoma in situ using WLIs. The diagnostic performance of the AI-assisted diagnosis was of high quality, and the AI could distinguish the tumor grade based on tumor color.

## Introduction

Bladder tumor diagnosis and operation was usually performed using cystoscope. Among cystoscopic findings for bladder tumor, the prediction of benign, malignancy was depended on the experience of the urologist^1^. At transurethral resection of bladder tumor (TURBT), an experienced urologist estimated presence of malignancy lesion and tumor grade, and decided whether to perform superficial resection or additional bladder muscle biopsy. However, it is difficult to differentiate between chronic non-specific inflammation (CNI) and carcinoma in situ (CIS) and between low and high grade among urothelial carcinomas, and it is difficult to distinguish even when mass is too small. The national comprehensive cancer network guideline recommended consideration of the second look TURBT if the necessary muscle biopsy is not performed or incomplete TURBT^2^. The existing white light image (WLI) cystoscope has limitations in effective identifying the characteristics for small bladder tumors, flat tumors such as CIS. To increase diagnostic accuracy, narrow band image (NBI) cystoscope and blue light cystoscope have been developed^3–5^. Based on accurate diagnosis performed through these enhanced cystoscopes, high-quality TURBT without misdiagnosis, under-staging, or incomplete resection is the most important factor in non-muscle invasive urothelial bladder cancer management^6^. However, still better diagnostic image modality was required for high-quality TURBT.

Tumor grade is an important factor in determining whether or not to perform a muscle biopsy. High grade bladder tumor and low grade bladder tumor are empirically classified according to the shape of the tumor (papillary vs. sessile). However, it is not always clearly differentiated according to the tumor shape, it is difficult to distinguish flat tumors, such as CIS, from benign inflammatory lesions such as CNI. For increasing representativeness and overcoming the limitation of reliance on empirical distinguishing of tumor grades, artificial intelligence (AI) that used in various fields including the medical field was introduced in urological endoscope field. There are still few studies in the field of urology, especially bladder cancer diagnosis using AI. With the development of deep learning, image recognition by AI proved high accuracy in diagnosis^7^. AI-based image diagnosis is a potential modality to improve bladder cancer detection. For clinical application of AI in bladder tumor diagnosis, it is necessary distinguishing between the tumor grades (high grade vs. low grade, CIS vs. CNI, etc.) as well as determining cancer and non-cancer, however there was no study have been reported.

AI is a model that predicts based on learned data, and authors considered shapes and color as learned data. Several studies using numerical data of color using red/green/blue (RGB) method^8^ have already been reported in other carcinomas^9–11^, and we also have reported the study using numerical color data for hypoechoic lesions of transrectal ultrasound^12^. Accordingly, the authors performed deep learning of AI by collecting WLI and NBI cystoscopic images of histopathologically confirmed benign and malignant lesions and confirmed the diagnostic performance and ability to identify tumor contours. And making color data based on the tumor contours identified by AI, it was analyzed whether the tumor grades were able to distinguished according to the color of the tumor.

## Results

### Bladder cancer diagnostic performance of AI-assisted diagnostic device

**Figure 1:**
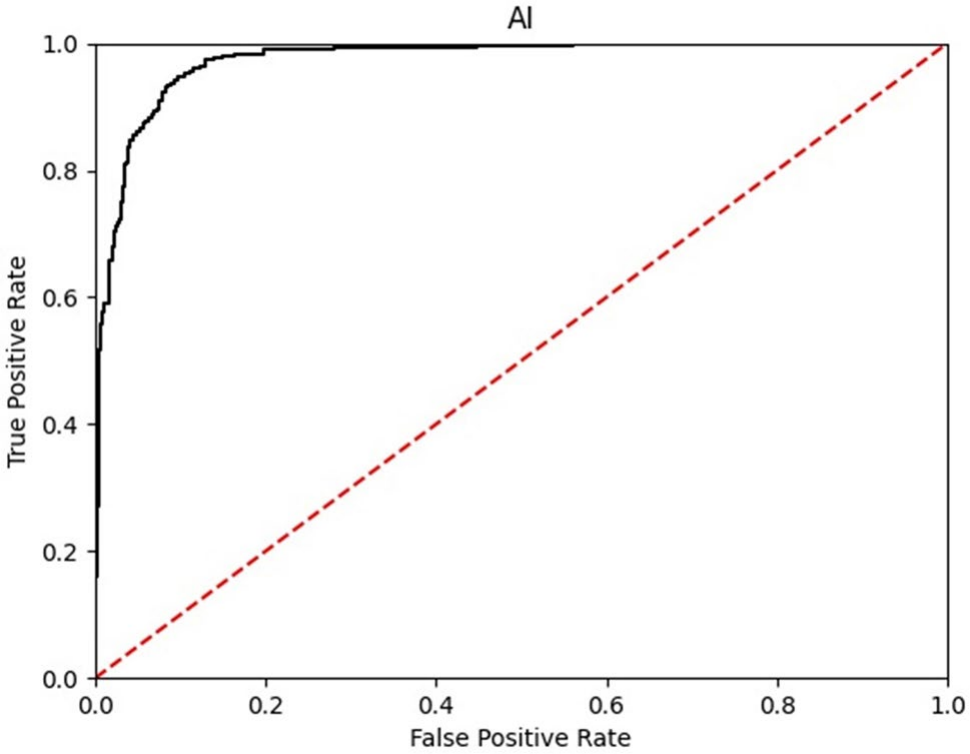
The AUC of AI-assisted diagnostic device. *AI* artificial intelligence, *AUC* area under the receiver operating curve.

The sensitivity of the AI-assisted diagnostic device was 95.0% (285/300 cases), the 95% confidence interval (CI) was 91.9–97.2, and the specificity was 93.7% (562/600 cases) and the 95% CI was 91.9–97.2. The diagnostic accuracy of the AI-assisted diagnostic device was 94.1% (847/900 cases), and the 95% CI was 92.4–95.6. The area under the receiver characteristic curve was 0.974 and cut-off value was 0.384 (Fig. 1). The accuracy of lesion location recognition of the AI-assisted diagnostic device was confirmed to be 74.7% for dice score coefficient (DSC) and 74.6–74.9 for 95% CI.

### Baseline RGB characteristics

We conducted the study only with anonymized cystoscopic images and histopathological pathology, so we could not identify other patient characteristics, including the patient's sex or laboratory values. The characteristics of RGB values according to benign tumor or tumor grade are presented in Table 1. In WLI, there were differences in red and blue values according to tumor grade (*p* < 0.001). In RGB values measured NBI or WLI by AI, there were no difference between benign vs. low grade and high grade, CNI vs. CIS. But between benign and low grade vs. high grade, there was a difference in red (*p* < 0.001) and blue (*p* < 0.001) values in WLI (Table 2).

**Table 1:** Baseline RGB characteristics according to tumor grade.

	Benign^a^	CNI	CIS	Low-grade	High-grade	*p*
**NBI**						
No	83 (4.6)	361 (20.1)	158 (8.8)	547 (30.4)	651 (36.2)	
Red	159.32 ± 25.32	157.27 ± 24.92	157.76 ± 23.48	160.75 ± 24.91	157.51 ± 25.87	0.168
Green	110.20 ± 24.91	115.05 ± 27.03	112.63 ± 21.59	115.59 ± 24.82	114.08 ± 23.16	0.294
Blue	84.63 ± 19.05	88.50 ± 20.26	86.50 ± 16.15	89.18 ± 19.33	87.89 ± 18.61	0.208
**WLI**						
No	198 (5.3)	729 (19.3)	256 (6.8)	1063 (28.2)	1522 (40.4)	
Red	180.35 ± 25.35	179.04 ± 22.81	180.85 ± 20.28	176.48 ± 24.39	184.62 ± 22.55	< 0.001
Green	129.38 ± 23.28	129.85 ± 19.29	131.73 ± 16.68	131.69 ± 22.34	130.10 ± 19.96	0.176
Blue	89.68 ± 32.86	94.45 ± 27.34	96.64 ± 25.52	90.94 ± 31.29	96.91 ± 28.62	< 0.001

Data are expressed as number (%), mean ± standard deviation.

*CIS* carcinoma in situ, *CNI* chronic non-specific inflammation, *NBI* narrow-band image, *RGB* red/green/blue, *WLI* white-light image.

^a^Benign defined cystitis cystica or cystitis grandularis except CNI.

**Table 2:** Baseline RGB characteristics in the NBI and WLI groups.

	Benign^a^	Low-and high-grade	*p*	Benign^a^, low-grade	High-grade	*p*	CNI	CIS	*p*
**NBI**									
No	83 (6.5)	1198 (93.5)		630 (49.2)	651 (50.8)		361 (69.6)	158 (30.4)	
Red	159.32 ± 25.32	158.99 ± 25.48	0.909	160.56 ± 24.95	157.51 ± 25.87	0.032	157.27 ± 24.92	157.75 ± 23.48	0.835
Green	110.20 ± 24.91	114.77 ± 23.93	0.094	114.88 ± 24.88	114.08 ± 23.16	0.550	115.05 ± 27.03	112.63 ± 21.59	0.278
Blue	84.63 ± 19.05	88.48 ± 18.94	0.074	88.58 ± 19.34	87.89 ± 18.61	0.513	88.50 ± 20.26	86.50 ± 16.15	0.231
**WLI**									
No	198 (7.1)	2585 (92.9)		1261 (45.3)	1522 (54.7)		729 (74.0)	256 (26.0)	
Red	180.35 ± 25.35	181.27 ± 23.66	0.598	177.09 ± 24.57	184.62 ± 22.55	< 0.001	179.04 ± 22.81	180.85 ± 20.28	0.262
Green	129.38 ± 23.28	130.75 ± 20.98	0.381	131.32 ± 22.50	130.10 ± 19.96	0.127	129.85 ± 19.29	131.73 ± 16.68	0.165
Blue	89.68 ± 32.86	94.45 ± 29.89	0.031	90.74 ± 31.53	96.91 ± 28.62	< 0.001	94.45 ± 27.34	96.64 ± 25.52	0.263

Data are expressed as number (%), mean ± standard deviation.

*CIS* carcinoma in situ, *CNI* chronic non-specific inflammation, *NBI* narrow-band image, *RGB* red/green/blue, *WLI* white-light image.

^a^Benign defined cystitis cystica or cystitis grandularis except CNI.

### Diagnostic performance of tumor grade by color measured by AI

**Figure 2:**
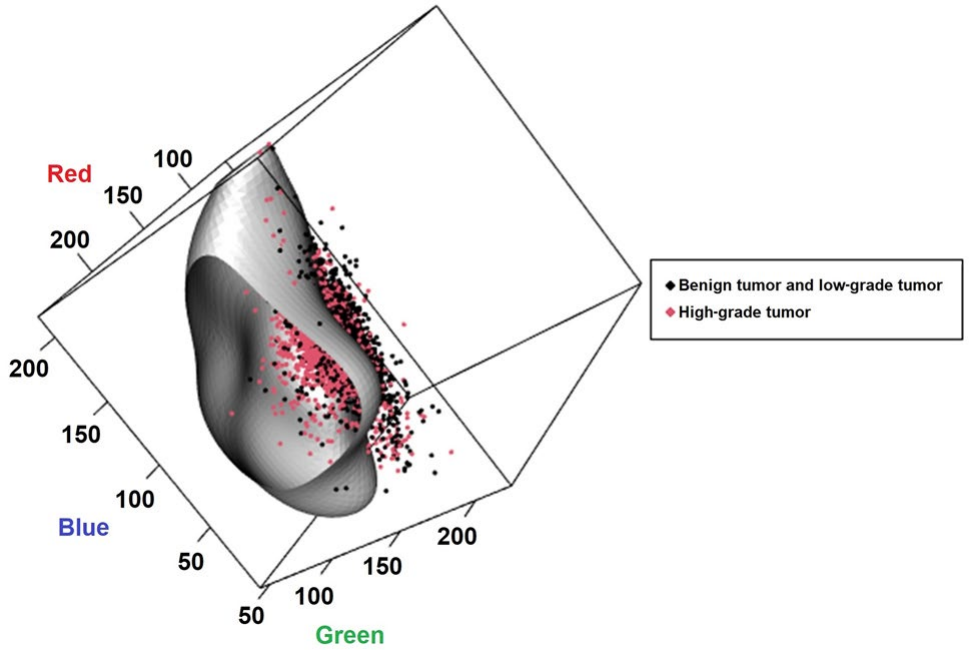
The separating hyperplane of the two groups using SVM in white light image cystoscope. *SVM* support vector machine.

The optimal cut-off value and diagnostic performance by support vector machine (SVM) was presented Table 3. Overall, the diagnostic performance was better in WLI than in NBI. In WLI, at diagnosis of benign vs. low grade and high grade, there were excellent results of 98% or more in both sensitivity and specificity and diagnostic accuracy. In distinguishing between CNI and CIS, both sensitivity, specificity, and diagnostic accuracy were more than 90% in WLI. In benign and low grade vs. high grade, although the diagnostic performance was relatively low, the separating hyperplane using SVM was classified two groups in WLI (Fig. 2).

**Table 3:** Diagnostic performance and differentiation of tumor grade according to tumor color by SVM.

	Cut-off value	AUC (95% CI)	Sensitivity (95% CI)	Specificity (95% CI)	Accuracy (95% CI)
**NBI**					
Benign^a^ vs. low-and high-grade	0.934	0.899 (0.863–0.931)	0.854 (0.834–0.874)	0.771 (0.681–0.861)	0.849 (0.829–0.868)
Benign^a^, low-grade vs. high-grade	0.507	0.583 (0.553–0.613)	0.644 (0.607–0.680)	0.478 (0.439–0.517)	0.562 (0.535–0.589)
CNI vs. CIS	0.298	0.890 (0.852–0.925)	0.873 (0.822–0.925)	0.834 (0.795–0.872)	0.846 (0.815–0.877)
**WLI**					
Benign^a^ vs. low-and high-grade	0.930	0.992 (0.984–0.999)	0.993 (0.990–0.997)	0.980 (0.960–0.999)	0.992 (0.989–0.996)
Benign^a^, low-grade vs. high-grade	0.535	0.701 (0.682–0.720)	0.670 (0.647–0.694)	0.629 (0.602–0.656)	0.651 (0.634–0.669)
CNI vs. CIS	0.251	0.972 (0.958–0.983)	0.980 (0.964–0.997)	0.912 (0.892–0.933)	0.930 (0.914–0.946)

Data are presented as median (interquartile range).

*AUC* area under the receiver operating characteristic curve, *CI* confidence interval, *CIS* carcinoma in situ, *CNI* chronic non-specific inflammation, *NBI* narrow-band image, *SVM* support vector machine, *WLI* white-light image.

^a^Benign defined cystitis cystica or cystitis grandularis except CNI.

## Discussion

We confirmed that the newly developed AI-assisted diagnostic device has good diagnostic performance and ability to identify tumor contours, and tumor grade was distinguished by colors inside tumor contour that recognized by AI. The AUC for benign and low-grade tumors vs. high-grade tumors was relatively low, however, it was distinguished by separating hyperplane using SVM in WLI. Our results help follow-up studies of cystoscopy using AI.

Cystoscopy is the gold standard for bladder cancer diagnosis. Imaging modalities such as abdominal pelvic computed tomography and bladder magnetic resonance imaging are also used to diagnose bladder cancer. These modalities are useful for determining whether bladder cancer has metastasized or invaded the muscle layer or beyond but have limitations in identifying small tumors in the early stages. Additionally, it is more challenging to diagnose bladder tumors if the bladder is empty. Several guidelines recommend the use of cystoscopy when bladder cancer is suspected. However, it is sometimes difficult to differentiate between benign and malignant bladder tumors, and the diagnosis of CIS is challenging. Bladder cancer is prone to recurrence and is characterized by multiple lesions. The accurate identification of tumor margins and complete TURBT are crucial in preventing recurrence. However, although NBI cystoscopy and blue-light cystoscopy can overcome the limitations of white-light cystoscopy, better diagnostic tools are needed. Our results demonstrated the potential of AI models for accurately diagnosing bladder cancer.

In the field of urology, prostate cancer is being extensively studied from diagnosis to treatment using AI^13^. However, in the field of cystoscopy using AI, to our knowledge, there are only six reports^14–19^. Chan et al. reported a review article on previous studies on bladder cancer diagnosis using AI^7^. This study reviewed 1 brief correspondence, 1 presentation, and 4 research papers. The training dataset volumes for these reports were between 1680 and 6658 images, and our AI model was trained 8244 images, more than in these previous reports. The combined sensitivity was 89.7% (95% CI 87.5–91.4%) and the integration specificity was 96.1% (95% CI 89.0–98.7%), the diagnostic accuracy was 85.6–96.9%. Only one study was identified that evaluated the ability of AI to identify bladder image using DSC^20^. The DSC of this previous study was an average of 0.67, which was slightly lower than our result, but there was a difference in evaluating the ability to identify bladder stones and ureteral orifice in addition to tumors. The results of our AI-assisted diagnostic device are similar to or slightly superior to those of previous reports.

Our study is valuable and scalable as it is the first study to bladder lesions using RGB method. There were some previous studies using RGB method to distinguish pulmonary nodule^21^, esophageal lesions^22^, and skin lesions^23^. However, there were no studies using RGB method in the field of cystoscopy, and no studies in the field of urology that machine-learned the average RGB inside AI-identified tumor contours so we could not comparable, but this also presented good results. We have reported a successful study by applying the RGB method in transrectal ultrasound^12^. The color of bladder mass is different from that of the normal bladder mucosa, we made numerical data that the color of bladder tumor through RGB method. In our previous study, RGB was calculated as the average of three points of hypoechoic lesion on transrectal ultrasound, but in this study, the average RGB value of the area inside the tumor contours recognized by AI was calculated. This improved the representativeness of the RGB values of tumor compared to our previous study.

Our results using RGB in WLI show superior performance in specificity than the results of conventional NBI, especially in distinguishing between CNI and CIS (0.912 [95% CI 0.892–0.933] vs. 0.768 [95% CI 0.730–0.802])^24^. NBI limited the optical spectrum used in cystoscope by Wlters which allow transmission of light at short wavelengths of 415 nm (blue) and 540 nm (green)^24,25^. This allows light to penetrate the bladder surface tissue and hemoglobin preferentially absorbs these wavelengths, increasing the visibility of capillaries and submucosal vessels. According to a previous meta-analysis, NBI presented higher diagnostic accuracy than WLI in humans^24^. However, our results presented a higher AUC in WLI. It is presumed that this is because the limited short wavelength of light limits the difference in intrinsic color according to the grade of the tumor.

We confirmed the effectiveness of the deep learned AI-assisted diagnostic device and at the same time confirmed that the tumor grade was able to classify according to the color of the tumor. Our study is one of the few studies that confirmed the usefulness of AI in cystoscopic image diagnosis and is the first study to classify tumor grades through machine learning of average RGB inside tumor contour recognized by AI. We wonder if better results will be obtained if the deep learned AI additionally learns RGB data of tumor color. The results of these additional studies will be able to check whether the variables that AI learned included color data. Our study is valuable as a pilot study in this field, it had many limitations. First, our study reports that the diagnostic performance is good, but the number of initially trained images may not be sufficiency in the deep learning field. By training more numbers and diverse types of images, higher diagnostic performance and more similar tumor contours identification expected. The DSC for tumor identification is 0.75, and the average RGB value was measured based on images with a higher concordance rate of tumor identification between the physician and AI, but the RGB value may still be different from the RGB value of the actual tumor. Again, if more images are trained, more accurate RGB values were obtained. Second, as our first experience to evaluate the color of the overall tumor, color normalization was not performed because the color space in rendered images would differ significantly from that in real images. Additional considerations for proper color normalization processing are required. Third, since only internal test was performed about diagnostic performance of AI, we need to confirm the results of our study with external validation. In addition, as the study was conducted on per-image-basis, the independence between data sets may have been lower than that if the study was conducted on a per-patient-basis, and this may have affected the results. The multicenter prospective study we are planning will consider this issues. And our color data were divided according to tumor grade, the number of cases was insufficient in some types, including benign lesion images. Therefore, we focused on training rather than training and validation of existing data. Because of this, the possibility of overtraining cannot be ruled out. Additional validation and test is required and may require further training with larger number of color data. Fourth, the initially trained images were prepared by a urologist, it is consistent, but there is a possibility that the lesion may have been missed or underestimated or overestimated. Finally, differences according to urine turbidity and the distance between the target and the endoscope were not considered.

AI is widely used in various medical fields, and in the field of urology, it is applied from diagnosis to treatment for prostate cancer. However, research in the field of cystoscope is still insufficient. We developed an AI-assisted diagnostic device that distinguishes cancer and benign lesions by identifying bladder tumor contour through deep learning and confirmed that tumor grades are classified using SVM according to the color of the tumors obtained thereby. Our study is still in its infancy, but we hope that it will serve as the foundation for the development of a real-time AI-assisted cystoscope in the future.

## Methods

### Ethic approval

This study was approved by the institutional ethics committee (Yonsei University Health System, Seoul, Korea; approval number: 3-2021-0287) and all procedures were conducted in accordance with the ethical standards of the 1964 Declaration of Helsinki and its later amendments. The informed consent requirement was waived by ethics committee because this study was based on retrospective, anonymous patient data and did not involve patient intervention or the use of human tissue samples.

### Images collection and annotated data set

We retrospectively collected data from 1010 consecutive patients who diagnosis bladder cancer by TURBT and 290 consecutive patients who diagnosis benign lesions by TURBT or diagnosis normal by cystoscopic exam between January 2017 and December 2020. Data were obtained from anonymized cystoscopic images and pathologic results. We collected 16,581 cystoscopic images of patients diagnosed with bladder cancer and 3957 cystoscopic images of patients diagnosed with benign lesions. All images were captured using flexible cystoscope (CYF-V2, Olympus Medical Systems Corporation, Tokyo, Japan). Among the acquired images, images of impacted stone in the bladder lesion, difficult to identify due to turbid urine or hematuria or out of focus, tumors except urothelial carcinoma or have no pathologic results were excluded. After screening, in order to secure more images of benign images, only images of clearly benign parts of cancer patients were additional extracted separately by experienced urologists. In the end, 905 patients with bladder cancer (6729 cancer images) and 405 patients with bladder cancer and without bladder cancer (4262 benign images) were classified, and a total of 10,991 images were learned and tested by AI. One urologist with more than 10 years of experience in TURBT identified tumor margins and differentiated between benign from malignant lesions by histopathological analysis. Only one image of each lesion was included in the analysis.

### AI-assisted diagnostic device and deep learning

We commissioned Infinyx Corporation to develop an AI-assisted diagnostic device (Robin-Cysto^®^, Infinyx, Daegu, South Korea) that automated analysis cancer and non-cancer through cystoscopic images. The images that we provided were divided into a deep learning set (10,091 images) and a test set (900 images, 300 benign images of 100 patients and 600 cancer images of 223 patients). The 300 benign images of 100 patients without bladder cancer used in the test set were extracted from the 290 patients without bladder cancer data set and tested. Among the images of patients with cancers, a benign image was not included in test set. The deep learning set was divided into the training set for the predictive model (8244 images, 2742 benign images and 5502 cancer images) and the validation set (1847 images, 1220 benign images and 627 cancer images) by Infinyx Corporation. The training, validation, and testing sets were classified on a per-image-basis.

**Figure 3:**
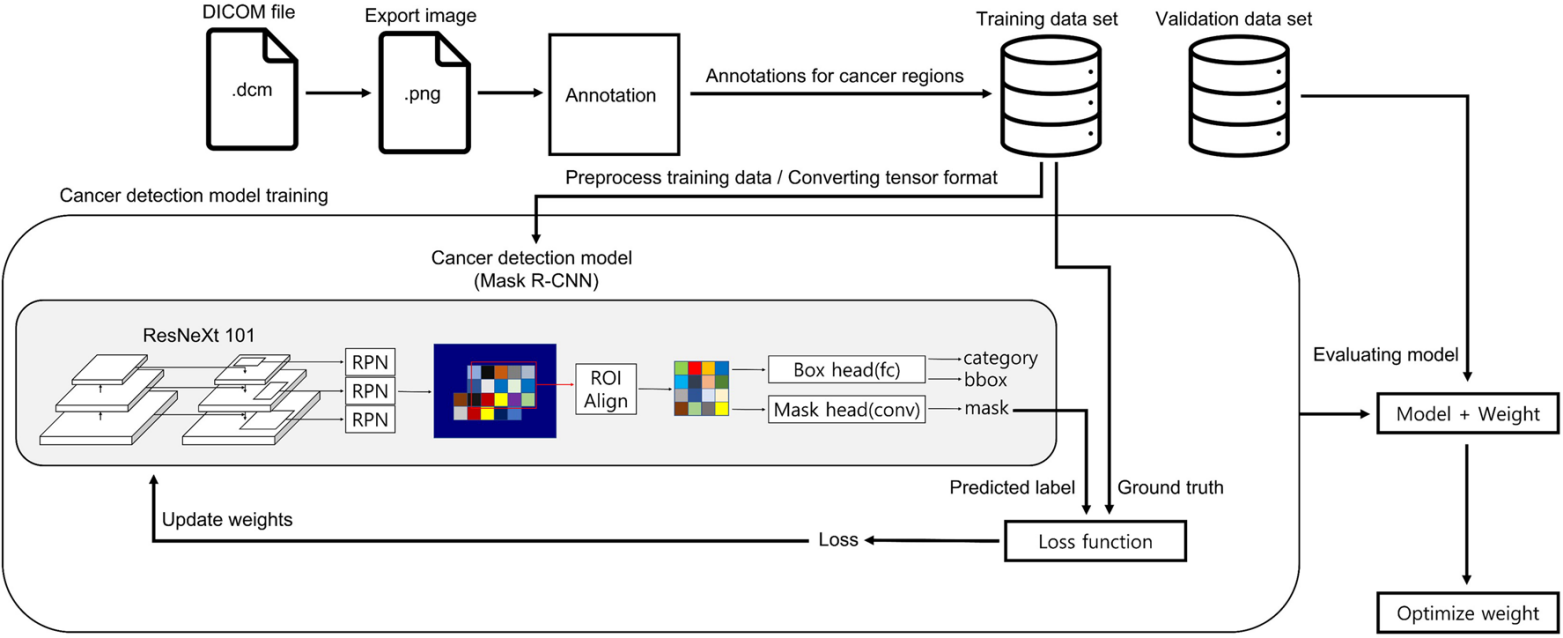
A schematic process for building an AI deep learning model. *AI* artificial intelligence, *bbox* bounding box, *Conv* convolutional layer, *DICOM* digital imaging and communications in medicine, *fc* fully connected layer, *Mask R-CNN* mask region-based convolutional neural networks, *ROI* resion of interest, *RPN* region proposal network.

**Figure 4:**
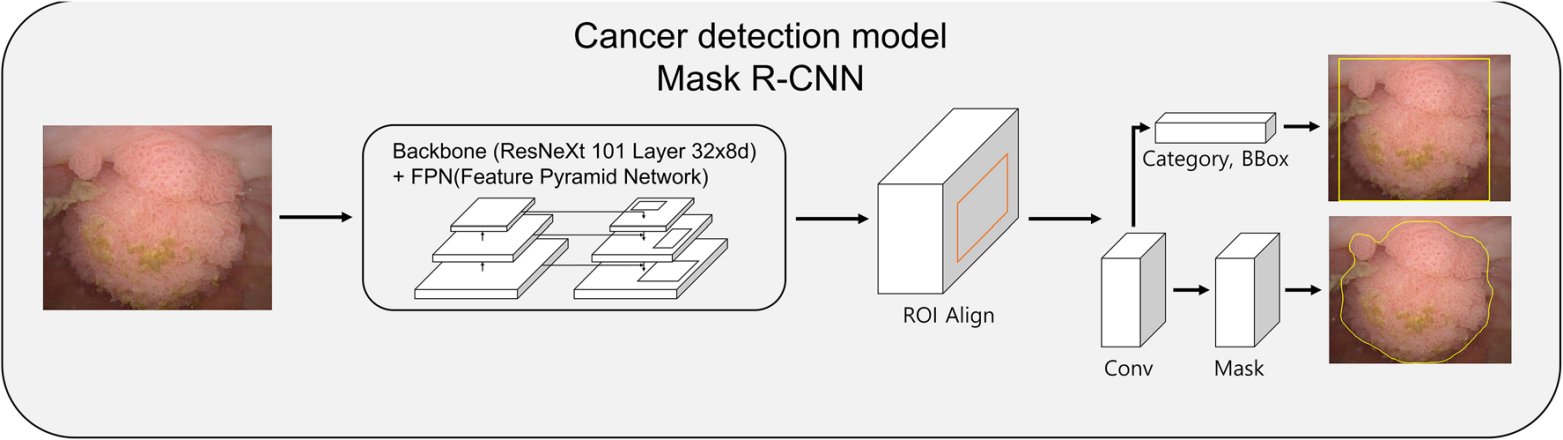
A practical example of the Mask R-CNN algorithm of an AI deep learning model. *AI* artificial intelligence, *bbox* bounding box, *Conv* convolutional layer, *Mask R-CNN* mask region-based convolutional neural networks, *ROI* region of interest.

The AI model consisted of a Mask RCNN with a ResNeXt-101-32 × 8d-FPN backbone. Feature maps were extracted from each layer using a feature pyramid network. For learning, image data was input into the AI model, and the AI model predicted the tumor contour. The AI model compared the real tumor area and the estimated tumor area using loss function, and the model weight was corrected using the extracted loss. With regard to loss functions, classification used log loss, bbox regression used smooth L1 loss, and mask learning used sigmoid cross-entropy loss for each pixel. Total loss was the sum of these three losses. The optimizer applied a momentum of 0.9 and a weight decay of 0.0001 to stochastic gradient descent, and the learning rate (LR) started at 0.001 with 30,000 steps and ended at 0.0001 with 70,000 steps. The accuracy was calculated by inputting the dataset to the AI model and the modified model weights, and the above process was repeated, and the weighted model with the highest accuracy was saved. The final training was conducted with 90,000 steps, and the batch size per step was 6 (Figs. 3, 4). The image size ratio remained unchanged during training, and image height was randomly resized to 480, 640, 720, and 1080 pixels. The code was used Python language (version 3.9.9.; Python Software Foundation, Wilmington, DE), and the PyTorch library was used.

### Diagnostic performance of AI-assisted diagnostic device

**Figure 5:**
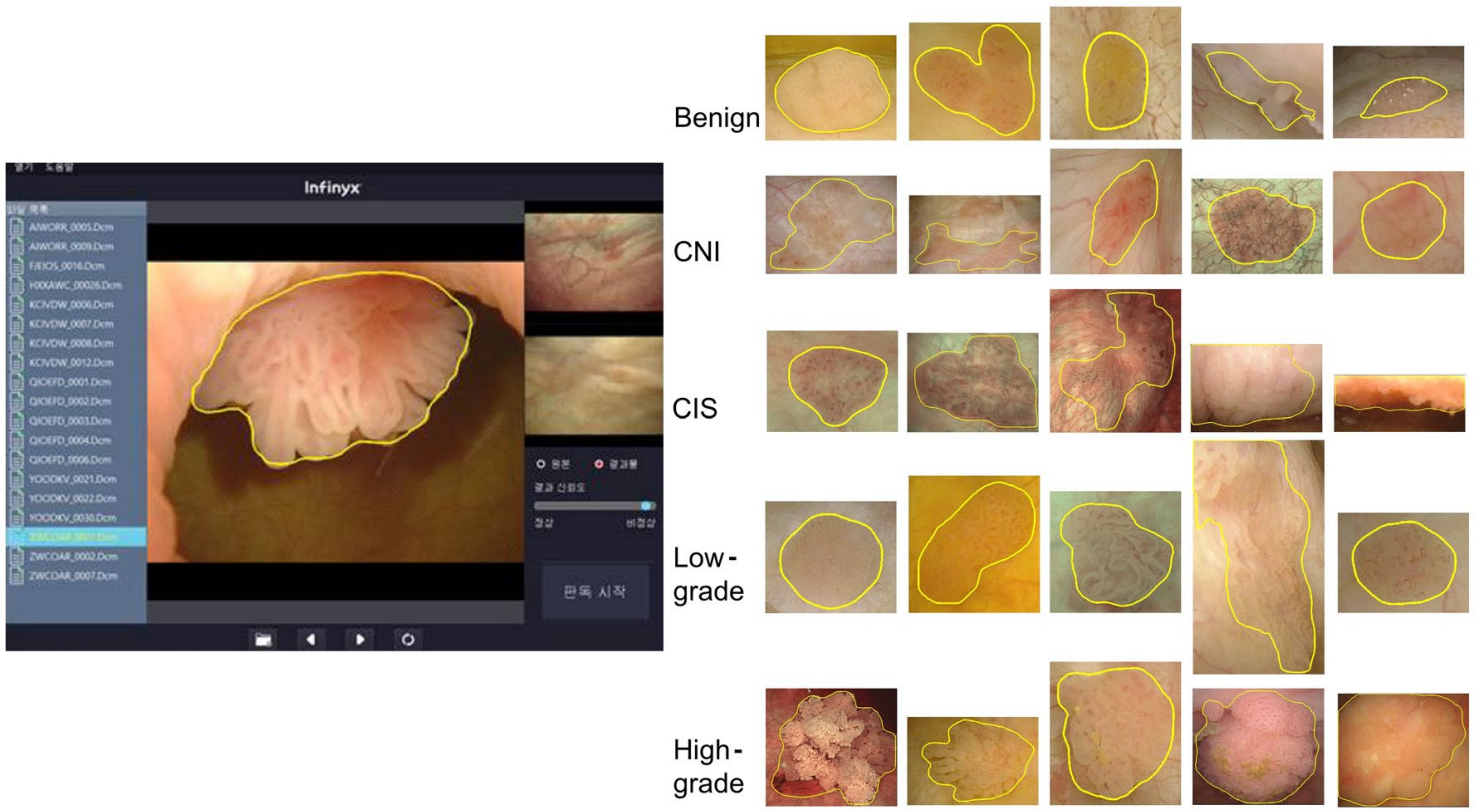
Visualization of bladder tumor contour recognized by AI. *AI* artificial intelligence, *CIS* carcinoma in situ, *CNI* chronic non-specific inflammation.

The deep learned AI reads the digital imaging and communications in medicine format file of the cystoscopy images, and visualizes the contour of bladder tumor on the image with bladder tumor (Fig. 5). Sensitivity, specificity, and diagnostic accuracy were verified with test set (cancer images: 600, benign images: 300), and the tumor area recognized by AI and the tumor area read by physician in the same image were compared with the DSC. The DSC quantifies the overlap between two images of manually segmented lesion and AI recognized lesion^26^.

### Data of tumor color

Among the 8244 images in the training set, color data were collected for the 5568 images in which the concordance rate of tumor identification by physician and AI were higher than DSC (cancer images: 4197, benign images: 1371). Using the embedded function of Robin-Cysto^®^, data of the color inside the tumor recognized by AI were obtained as the average RGB value from all pixels in the image^8^.

### Study endpoint

The end points were evaluation of diagnostic performance and tumor recognition ability through internal verification of the AI-assisted diagnostic device, and confirmation of difference in tumor grade according to the color inside the tumor contour identified by AI.

### Statistical analysis

The distinguishing ability of tumor grade using the RGB value was evaluated by confirming the optimal cut-off value of the prediction probability using the SVM model. SVM is a branch of machine learning for classification that learns the optimal "separation" between the characteristics of each group. This training algorithm finds a separating hyperplane with a maximum margin defined as the distance from each side to the nearest data point^27^. The optimal cut-off value was based on the Youden index (sensitivity + specificity − 1). In the case of three groups, the cut-off value was not able to confirmed because the probability of belonging to each group was calculated, so benign and cancer lesions were divided into two groups. Continuous variables are expressed as the mean ± standard deviation or median (interquartile range). Categorical variables are reported as number and frequency. Divided two groups were compared using the independent t-test for continuous variables. The results of the ability to classify tumor grade according to RGB values were presented as area under the receiver operating characteristic curve, sensitivity, specificity, and diagnostic accuracy. Statistical analyzes were performed using R Statistical Package (version 4.1.1.; Institute for Statistics and Mathematics, Vienna, Austria). Statistical significance was set at *p* < 0.05.

## Data Availability

The datasets used and analyzed during the current study are available from the corresponding author upon reasonable request.
